# In silico identification of critical proteins associated with learning process and immune system for Down syndrome

**DOI:** 10.1371/journal.pone.0210954

**Published:** 2019-01-28

**Authors:** Handan Kulan, Tamer Dag

**Affiliations:** Computer Engineering Department, Kadir Has University, Istanbul, Turkey; Zapadoceska univerzita, CZECH REPUBLIC

## Abstract

Understanding expression levels of proteins and their interactions is a key factor to diagnose and explain the Down syndrome which can be considered as the most prevalent reason of intellectual disability in human beings. In the previous studies, the expression levels of 77 proteins obtained from normal genotype control mice and from trisomic Ts65Dn mice have been analyzed after training in contextual fear conditioning with and without injection of the memantine drug using statistical methods and machine learning techniques. Recent studies have also pointed out that there may be a linkage between the Down syndrome and the immune system. Thus, the research presented in this paper aim at in silico identification of proteins which are significant to the learning process and the immune system and to derive the most accurate model for classification of mice. In this paper, the features are selected by implementing forward feature selection method after preprocessing step of the dataset. Later, deep neural network, gradient boosting tree, support vector machine and random forest classification methods are implemented to identify the accuracy. It is observed that the selected feature subsets not only yield higher accuracy classification results but also are composed of protein responses which are important for the learning and memory process and the immune system.

## Introduction

Down syndrome (DS) is a very common identifiable genetic cause of intellectual disability (ID) and affects approximately one in 700 live births [1]. In addition to ID, people with DS are at risk for certain types of blood diseases, like leukemia, autoimmune disorders and Alzheimer’s disease (AD) [2, 3].

The characteristics of DS can be diagnosed by the observation of the extra copy of whole or a portion of the long arm of human chromosome21 (Hsa21). Hsa21 is responsible for nearly 160 protein-coding genes and five microRNAs [4]. Over expression of these proteins which include transcription factors, cell surface receptors, protein modifiers, adhesion molecules, RNA splicing factors and components of many biochemical pathways can cause the learning and memory (L/M) deficits. In addition for a person diagnosed with DS, the number of neurons and cellular morphology are not normal in brain regions, such as the cortex, cerebellum and hippocampus [5–7].

Scientists have been using mice to find a treatment for the DS. However, it is compelling to model DS in mice since orthologs of the Hsa21 genes map to many mouse chromosomes, chromosomes 10, 16 and 17. However, Ts65Dn trisomic mice consisting 88 orthologs of Hsa21 protein coding genes and 5 microRNA genes can be used as a DS mouse model [8, 9]. For the treatment of the DS, many efforts are in progress in order to develop drugs. More than 20 drugs which have diverse properties, such as N-methyl-D-aspartate receptor (NMDAR) antogonist, *γ*− aminobutyric acid A (GABAA) receptor antagonists, acetylcholinesterase inhibitors and the green tea component have been shown to be effective for rescuing performance in L/M tasks [10–18].

One of these drugs called memantine, is an NMDAR antagonist and it modulates excitatory neurotransmission through antagonizing the activity by binding the N-methyl D-aspartate 2A (NR2A) and N-methyl D-aspartate 2B (NR2B) subunits with high on and off rates [13, 19, 20]. The NMDARs gated by glutamate plays an essential role in excitatory transmission and L/M process. When excessive amounts of glutamate binds to NMDARs, they generate free radicals and cause the synaptic dysfunction [21]. However, when memantine binds to NMDARs, it prevents glutamate binding and thus prevents cognitive and memory deficits [22]. By inspecting the protein profiles of normal and trisomic mice with and without memantine treatment, the impact of memantine on learning capability can be evaluated. In order to understand which protein expressions in control mice are important for successful learning, which abnormalities for Ts65Dn trisomic mice cause failed learning, and which changes by memantine give rise to rescued learning for Ts65Dn trisomic mice, protein expression data has been evaluated by computational learning methods [23, 24].

In this study, we applied supervised learning methods to protein expression data for 77 proteins (thus a 77dimensional space) taken from the cortex of control and Ts65Dn trisomic mice, with and without memantine treatment and with and without contextual fear conditioning (CFC). We compared our results with previous studies where Self Organizing Map (SOM) was used to pinpoint functional or regulatory similarities among proteins with similar expression profiles.

In previous works, it was shown that discriminating proteins were enriched in processes, such as mTOR signaling pathway, AD, MAPK signaling pathway and apoptosis [25]. In addition, it was also stated that DS could be related to the immune system and considered the DS as an immune disorder [26]. It was shown that interferon response which happened in response to the presence of several pathogens, such as parasites, viruses, bacteria and also tumor cells were consistently activated in cells obtained from individuals with the DS and could cause autoimmune disorders and leukemia, and perhaps AD. Because of this reason, we have inspected the feature subsets to understand their role in the immune system.

Per our findings, the selected protein subsets that we found can result in more accurate classification models of mice than those selected protein subsets chosen in previous studies. We have achieved better results by using different preprocessing steps and feature selection methodology when compared to previous studies. To select the best parameters for different classification methods to differentiate control and Ts65Dn trisomic mice, we applied the grid search method. To build a robust and reliable classification model, cross validation is used. Thus, our results are not only more accurate, but also composed of protein expressions that are important in the L/M process and the immune response. We made these conclusions after inspecting the literature to understand the importance of proteins in selected feature subsets. As a consequence, we believe that the protein subsets selected by applying the method described in this paper can be utilized to understand the effects of proteins on L/M task and can be used to develop effective drugs.

### Related work

Protein abnormalities in DS were studied by using different techniques to select proteins in the literature as stated in Table 1. Firstly, Ahmed et al [27, 28] studied a three level mixed effects (3LME) statistical analysis model of the Ts65Dn trisomic and normal mice protein profiles with and without exposure to CFC. Later, Higuera et al [25] examined the profiles using unsupervised learning, SOM to find important proteins for three cases; successful learning, rescued learning with memantine and failed learning. However, Eicher et al [29] believed that the problem was more suitable as a classification problem instead of a clustering problem and applied the linear SVM to find out proteins are discriminatory between two classes or groups of classes. A. Block et al [12] applied 3LME and used another drug RO4938581 for rescuing protein anomalies. B. Feng et al [30] used adaptive boosting (AdaBoost) method for feature selection and applied random forest, SVM and decision tree classification techniques for differentiating normal and trisomic mice.

**Table 1:** Studied techniques in the literature.

	Techniques
Ahmed et al [27]	3LME statistical model
Higuera et al [25]	Unsupervised Learning(SOM)+Wilcoxon rank-sum test
Eicher et al [29]	Supervised Learning(Linear SVM)+Wilcoxon rank-sum test
A. Block et al [12]	3LME statistical model
B. Feng et al [30]	Supervised Learning(AdaBoost)

Ahmed et al [27, 28] measured 84 protein expression levels in the hippocampus and the cortex of normal mice to evaluate learning capability. Context shock (CS) and shock context (SC) classes were partitioned into memantine or saline injected subclasses yielding four different classes and these four classes were analyzed. Memantine usage improved L/M capability in patients with AD [22]. Thus, the effects of memantine were assessed for comparison with DS. They showed that more than half of the protein levels changed significantly in the hippocampus. The number of proteins showing important changes in the cortex was smaller [27]. Furthermore, they applied this study to Ts65Dn trisomic mice to understand their protein dynamics for learning capabilities. They showed that there were indicative differences between the normal and Ts65Dn trisomic mice profiles [28].

Higuera et al [25] claimed that the statistical analysis performed by Ahmed et al [27, 28] was not satisfactory to determine all changes in protein profiles. They proposed that machine learning methods might fulfill these needs. They applied SOM to cluster protein profiles by using 77 proteins rather than 84. They described a set of class-specific clusters which were constituted from a set of adjacent nodes containing only samples from a single class or a node with at least 80% of its samples obtained from one mouse. Then, they applied the Wilcoxon rank-sum test and detected that protein levels were significantly different between each pair of clusters and specified those proteins as discriminatory between two classes.

A. Block et al [12] used *GABA*_A_*α*5− selective modulator, RO4938581, for rescuing protein anomalies of trisomic Ts65Dn mice. In their work, 91 protein levels relevant to brain functions were measured by applying the 3LME. 44 of the 52 anomalies in trisomic Ts65Dn mice were corrected by RO4938581.

Eicher et al [29] believed that the problem was naturally related to classification problem rather than clustering problem since the determination of proteins that can separate two classes or groups of classes was required. In addition, they stated that classification methods could give higher accuracy than clustering methods as re-labeling clusters might lower the result accuracy and also accuracy could be measured more efficiently by using quantitative methods like cross validation, training and testing prediction rather than a visual basis. Therefore, they applied linear SVM for differentiating proteins. The classification performance of the linear SVM algorithm was better than the methods used in previous studies. However, for determining important proteins for more than two classes as an input to Higuera et al [25], Eicher et al [29] aggregated classes to constitute new positive and negative classes. These aggregated class results are not compared with the Higuera’s work efficiently. Since linear SVM was not efficient for multi-class classification of proteins, multiclass classification methods were needed.

B. Feng et al [30] reduced feature subset from 77 to 30 features by applying AdaBoost method and applied Random Forest, Decision Tree and SVM classification methods to distinguish normal and trisomic Ts65Dn mice. They showed that selected protein datasets gave higher classification results. However, they did not consider control and Ts65Dn mice, with and without memantine treatment and with and without CFC stimulation subgroups. They were able to only differentiate control group from the trisomic group. Thus, their work did not show systematic analysis which was carried out with Higuera’s work by inspecting the subgroups. Also, AdaBoost has been a very efficient method for solution of the two-class classification problem. However, in going from two-class to multiclass classification, naive AdaBoost algorithm has restricted to the reduction of the multiclass classification problem to multiple two-class problems. Thus, multiclass classification algorithms are needed to determine which proteins are discriminatory when there are more than two classes. For this reason, using naive Bayes learner which is one of the machine learning algorithms for multiclass classification [31, 32], we applied forward feature selection technique in our previous work [33] for the determination of important proteins for the DS. After selecting features, DNN, random forest and SVM classification methods are used to differentiate control and trisomic Ts65Dn mice. The accuracy result of our work turned out to be higher than B. Feng’s work for all classification methods.

In this study, naive Bayes learner in forward feature selection method is used for learning process and features are selected based on their effects of the improvement in our model. After selecting features, DNN, gradient boosted tree, random forest and SVM classification methods are used to differentiate control and trisomic Ts65Dn mice. The control and Ts65Dn mice with and without memantine treatment and with and without CFC stimulation subgroups are analyzed and the accuracy results of different classification methods are compared with the accuracy results of feature subsets selected in Higuera’s work in which systematic analysis was carried out by implementing SOM for three cases, successful learning, rescued learning and failed learning.

## Materials and methods

### Datasets

The dataset that we used in this paper are obtained from University of California Irvine Machine Learning Repository [34]. The same data was also used in Higuera’s work [25] with which we will compare our results. The data contains of the expression levels of 77 proteins obtained from the nuclear fraction of cortex. In the dataset, there are 38 control mice and 34 trisomic Ts65Dn mice. 15 samples (three replicates of a five-point dilution series) are extracted from each mouse, resulting in 1080 samples.

The dataset is divided into eight classes of mice based on the protein profiles of 77 proteins after training in CFC with and without injection of memantine. These 77 proteins have roles for brain function, structure or development. Table 2 describes format of dataset in which rows show the individual mice and columns show the expression levels of the 77 proteins and the class of each mice.

**Table 2:** Description of protein expression data.

Mice	P1	P2	‥	‥	‥	P77	Class
mouse 1	0.504	0.747				1.676	c-cs-m
mouse 2	0.515	0.689				1.744	c-cs-m
mouse 3	0.509	0.730				1.926	c-cs-m
‥							
mouse n							

Table 3 shows eight classes of mice in the dataset based on their types, their exposure to CS or SC, applied drugs, number of mice in each class and their learning outcomes.

**Table 3:** Classes in the dataset.

Class	Type of Mice	Type of Experiment	Treatment	Number of Mice	Learning Outcome
c−SC−s	Control	Shock Context	Saline	9	No Learning
c−SC−m	Control	Shock Context	Memantine	10	No Learning
c−CS−s	Control	Context Shock	Saline	9	Normal Learning
c−CS−m	Control	Context Shock	Memantine	10	Normal Learning
t−SC−s	Trisomic	Shock Context	Saline	9	No Learning
t−SC−m	Trisomic	Shock Context	Memantine	9	No Learning
t−CS−s	Trisomic	Context Shock	Saline	7	Failed Learning
t−CS−m	Trisomic	Context Shock	Memantine	9	Rescued Learning

In CFC protocols [35], CS group are placed in a cage, waiting several minutes to explore the context. Later, an electric shock is applied. It is expected from wild type mice to link the context with an electric shock and would freeze after re-exposure to the same cage. The SC group is placed in a cage for controlling the effect of the shock alone. After placement in the cage, the electric shock is given immediately. It is expected that wild type mice do not learn to link the cage with shock and do not freeze after re-exposure the same cage. However, the trisomic Ts65Dn CS group of mice can not to learn and they do not freeze. However, if the Ts65Dn CS group of mice is injected with memantine, learning can be rescued [13].

After determining the groups, protein expression levels of each mice are measured with reverse phase protein arrays (RPPA) [36] which provides a quantitative analysis of the differential expression of proteins.

### Data preprocessing

For some of the mice in the dataset, one or more protein level measurements have missing values. The missing values are replaced by the average expression levels of the corresponding sample of the mice in the same class. For example, if a mouse is missing the first sample expression level information, the missing value is replaced by the average value of the first sample protein expression of other mice in the same class.

The replacement method that we use is different from previous studies. In the previous studies, missing values were replaced with the average value of all protein expression levels in same class mice. 15 tissue samples that are three replicates of a five-point dilution series were obtained per mouse. We considered the effect of dilution ratio and applied different calculations to handle missing values. In addition to replacing the missing parts, all measurements are normalized with Z-score normalization to prevent proteins with higher values influence on the classification result erroneously. In order Z-score normalization to preserve range (maximum and minimum), we applied Z-score normalization rather than max-min normalization which was applied in Higuera’s work [25]. With Z-score normalization as shown in Eq (1), mean of the scores is subtracted from each score and then divided into the standard deviation [37].

$$Z=\frac{x-\mu }{\sigma },$$ (1)

### Feature selection

Before building a classification model, dimensionality reduction is very crucial for the understanding the information about the class. Dimensionality reduction is the process of decreasing the number of features for identification of the most relevant and important variables. It has the effect of decreasing the computational cost. For dimensionality reduction, feature selection and feature extraction methods can be used. Feature selection choses a subset of features, while feature extraction generates a new feature set of original features.

The feature selection method, named as forward feature selection, is used in our work. It is the heuristic method which tries to find the optimal feature subset by iteratively selecting features based on the classifier performance. It begins with an empty feature subset and adds one feature at a time for each round. This one feature is taken from the pool of all features that are not in the feature subset and when added it results in best classifier performance. The above process is repeated until the required number of features are added. It does not examine all possible subsets and does not give a guarantee to find the optimal subset. However, it reduces the search time when compared to exhaustive feature selection [38].

In this study, forward feature selection is applied [39] using the Knostanz Information Miner (KNIME). The logic of program is a search loop. Inside the loop, the dataset is divided into a learning set (70%) and a validation set (30%). Learning set is used for the construction of the model in the current selection of the variables and validation set computes an unbiased error rate estimation. For the learning process, naive Bayes learner which was applied to multi classification problem is used. In spite of the underlying simplifying assumption of conditional independence, naive Bayes performs well with more than two classes problem. [33, 40]. In previous studies, the applied algorithms suffered from an efficient multiclass classification technique. In our studies, we eliminated this deficiency with naive Bayes algorithm in forward feature selection method.

## Classification methods

After selecting features, classification methods are applied for differentiating mice in different subgroups. We carried out four classification methods, DNN, gradient boosted tree, random forest and SVM. These classification methods are implemented by using Python and Scikit Learn package [41]. In order to select the most appropriate parameters of classification methods, grid search method [42] is applied. Also, for building robust and reliable classification model, 5 fold cross validation is applied. Thanks to cross validation, a learner can generalize to an unknown data set. In K Fold cross validation [43], the data is partitioned into k subsets. Only one of these subsets is used as the test set and the others are constituted to a training set at each time. This procedure is repeated k times. The error estimation is averaged over all k trials to get total effectiveness. This way significantly decreases bias since we are using most of the data for fitting. It also significantly reduces variance as most of the data is also being used in validation set. In the rest of this section, a brief discussion on the four types of classification methods that we have used in our study is described.

### Deep Neural Network

DNN is type of a neural network with multiple layers between the input and output layers. Neural networks are inspired from human brain as they acquire knowledge through learning and composed of connected units called artificial neurons which is analogous to biological neurons in a brain. Each connection between neurons can transmit a signal to another neuron and may also have a weight that can increase or decrease the strength of the signal. Neurons are generally organized in layers and signals travel between layers. In order turn the input into the output, DNN tries to find the relationship whether linear or not. The network moves through the layers calculating the probability of each output [44].

### Gradient boosted tree

Boosting is a sequential ensemble method that converts weak learners to a strong learner by promoting previously mislabeled data with higher weight. Thus, the subsamples of data have an different probability of appearing in subsequent models and the ones with the highest probability of error appear most [45]. Gradient boosting builds the model in a sequential way. At each step the decision tree *h*_*m*_(*x*) that is base learner is selected to minimize a loss function L given the current model *F*_*m*−1_(*x*) as shown in Eq (2).

$$\begin{array}{c} \begin{array}{c} F_{0}\left(x\right)=argmin\sum_{i=1}^{n}\left(L\left(y_{i}\right),\gamma \right),\\ F_{m}\left(x\right)=F_{m-1}\left(x\right)+\gamma h_{m}\left(x\right) \end{array} \end{array}$$ (2)

In above equation, m is the number of iterations, *F*_*m*_(*x*) is the model and *γ* is the learning rate.

### Support vector machines

SVM is a supervised machine learning classification method which uses a data set d-dimensional Euclidean space. The number of d represents the number of features in the data set. Later, SVM finds an optimal (d-1)dimensional hyperplane as given in Eq (3) to separate the data by class. In this equation, w represents a weight vector of length d and b represents a bias term. The distance between the hyperplane and the nearest data point from either part of the hyperplane is known as the margin. In order to classify new data correctly, the distance between between the hyperplane and any point within the training set must be higher [46].

w.x+b=0 (3)

### Random forest

**Fig 1:**
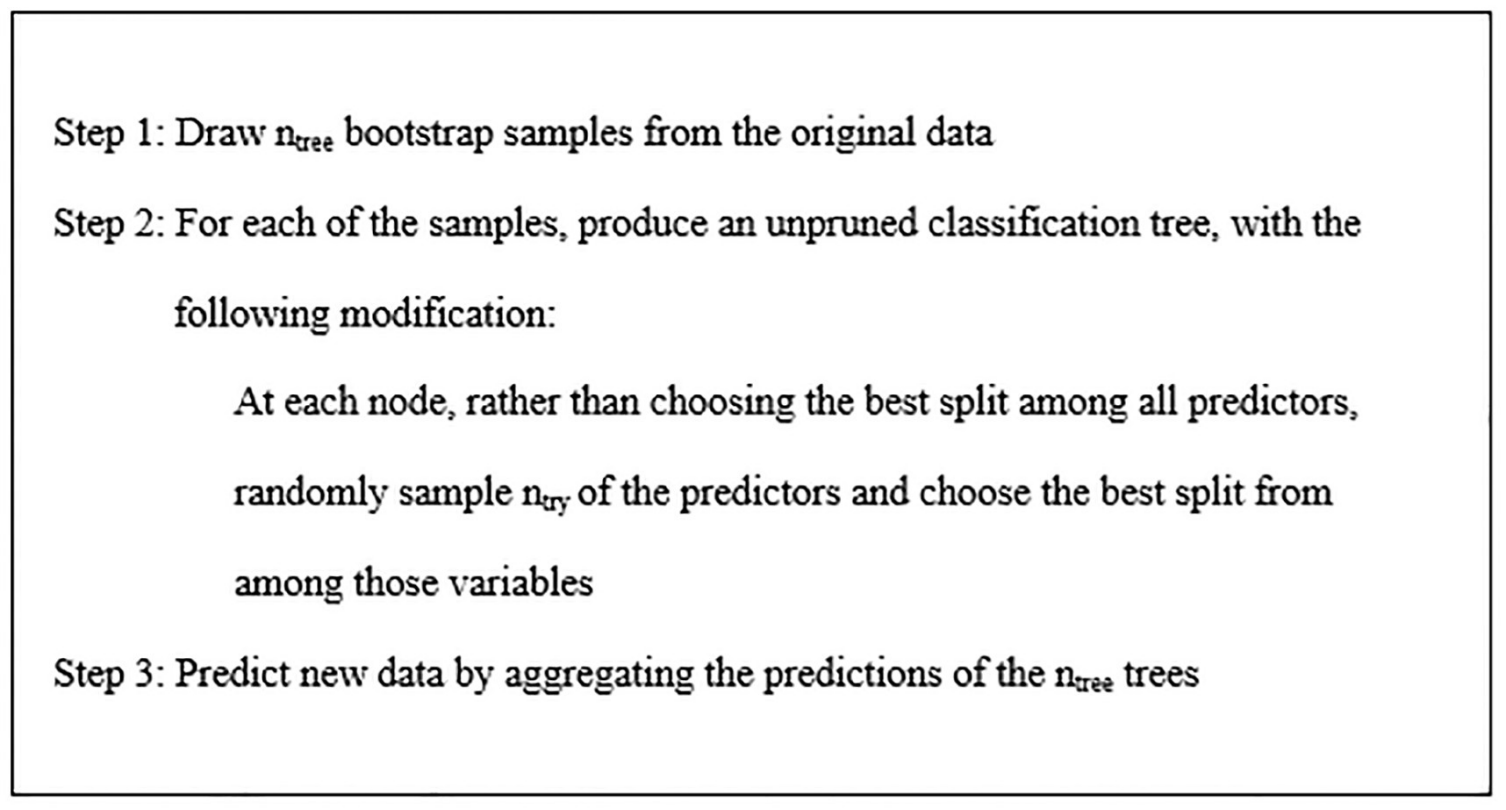
Random forest classification algorithm.

Random forest is composed of many decision trees which are selected from a random subset of training set. It constructs random forest by combining a large number of decision trees and outputs the class that is the mode of the classes or mean prediction of the individual trees [47]. Random forest classification methodology is described in Fig 1.

Model is tuned with two parameters ntree and ntry to get optimized forest architecture. The parameter ntree specifies how many trees are to be built to populate the random forest where as ntry specifies the number of variables that will be considered at any time in deciding how to partition the dataset.

## Results

Using the KNIME tool [39], forward feature selection technique is used to obtain the feature subsets for identifying the critical proteins in successful learning, rescued learning and failed learning cases. Afterwards, in order to validate importance of selected proteins, principal component analysis (PCA) is carried out. After determination and validation crucial proteins, DNN, gradient boosted tree, random forest and SVM classification methods are executed. PCA and application of classification methods are carried out with Python and Scikit learn package [41]. Also, grid search which is the parameter optimization technique [42] and 5 fold cross validation are done for obtaining robust and reliable classification results. The below subsections successively show the results of feature selection method, PCA and classification methods for successful learning, rescued learning and failed learning.

### Feature selection results

Forward feature selection method is applied with KNIME tool [39] and then results of selected feature subsets are compared with Higuera’s work [25]. In that work, three feature subsets were highlighted for normal learning, rescued learning and failed learning. Higuera et al [25] analyzed control mice and trisomic mice separately and together in order to understand the changes in protein levels. To understand which of the protein expression level changes are required for successful learning, all groups of normal mice were inspected in the first case. To determine important proteins in rescued learning, trisomic mice exposed to CFC with and without memantine were analyzed in the second case. The third case found out important protein abnormalities in failed learning by comparing normal and trisomic mice protein expression levels.

In our work, similar to Higuera’s work, we also selected three feature subsets to understand critical proteins in normal learning, rescued learning and failed learning. The number of features in feature subsets are selected based on the number stated in Higuera’s work [25] for comparison purposes. The difference in preprocessing and feature selection methods affect results in a positive manner and important proteins that were not highlighted in the previous work are found in normal learning, rescued learning and failed learning cases.

#### 1. Feature subset of data from control mice

Table 4 shows the selected features and their accuracy with normal learning when selected feature is added to the subset. Under the first case, feature subset is selected from control group mice data. By comparing control group mice with and without memantine treatment and with and without CFC stimulation, critical proteins in successful learning can be understood. When compared with Higuera’s work [25], there are 4 common proteins (SOD1, pGSK3B, S6, CaNA) out of 11 proteins which are shown in bold. After literature review, it can be deduced that the selected proteins in successful learning are related to the L/M pathway and the immune responses [48–62].

**Table 4:** Feature subset of normal learning.

Feature No	Accuracy of Feature	Feature Subset	Feature Subset of Previous Work [25]
1	0.656	**SOD1**	DYRK1A
2	0.751	Ubiquitin	ITSN1
3	0.852	**pGSK3B**	pERK
4	0.873	**S6**	BRAF
5	0.905	**CaNA**	**SOD1**
6	0.921	IL1B	pNUMB
7	0.937	BAX	**pGSK3B**
8	0.942	pNR2A	CDK5
9	0.942	BDNF	**S6**
10	0.942	pJNK	GFAP
11	0.942	pCFOS	**CaNA**

#### 2. Feature subset of data from trisomic Ts65Dn mice

In the second case, to understand the important proteins in rescued learning, features are selected from data consisting of trisomic mice which are exposed to CFC with and without memantine. When exposed to CFC, the trisomic mice fail to learn if they are not treated with memantine which rescues the learning performance. Table 5 shows the selected features and their accuracy results obtained when the corresponding feature is added to feature subset in rescued learning case. There are 2 common proteins (BRAF, CDK5) when compared with previous work. Literature search shows us that selected proteins in rescued learning are related to L/M process and immune response [63–69].

**Table 5:** Feature subset of rescued learning.

Feature No	Accuracy of Feature	Feature Subset	Feature Subset of Previous Work [25]
1	0.762	**BRAF**	DYRK1A
2	0.838	S6	pERK
3	0.85	**CDK5**	**BRAF**
4	0.887	BDNF	**CDK5**
5	0.887	pCREB	RRP1
6	0.9	PKCA	GFAP
7	0.912	SOD1	GluR3
8	0.925	PSD95	P3525
9	0.925	pNR2A	Ubiquitin

#### 3. Feature subset of data from control and trisomic Ts65Dn mice

Under the third case, for identifying proteins that are critical in failed learning with trisomic mice, features are selected from protein expression levels of trisomic mice exposed to CFC without memantine and protein expression levels of control mice which are exposed to CFC with and without memantine. Table 6 shows the selected features and accuracy results of feature subset in failed learning. There are 2 common proteins (P38, GluR3) out of 10 proteins when compared with former work. Selected proteins in failed learning play important roles in signaling pathway [70, 71].

**Table 6:** Feature subset of failed learning.

Feature No	Accuracy of Feature	Feature Subset	Feature Subset of Previous Work [25]
1	0.636	**P38**	pNR1
2	0.713	pPKCAB	APP
3	0.775	CAMKII	MTOR
4	0.814	pCAMKII	**P38**
5	0.868	**GluR3**	NR2B
6	0.891	DSCR1	RAPTOR
7	0.907	nNOS	S6
8	0.915	BAX	Tau
9	0.93	pCFOS	**GluR3**
10	0.93	ERK	EGR1

### Validation of selected proteins subsets

**Fig 2:**
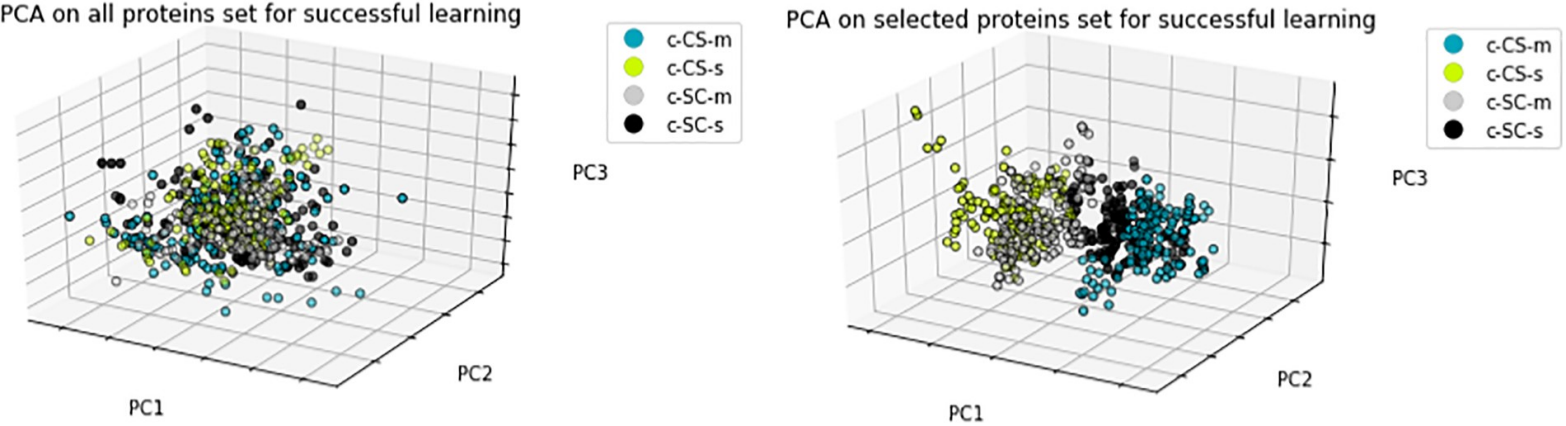
PCA of all proteins set and selected proteins subset for successful learning.

**Fig 3:**
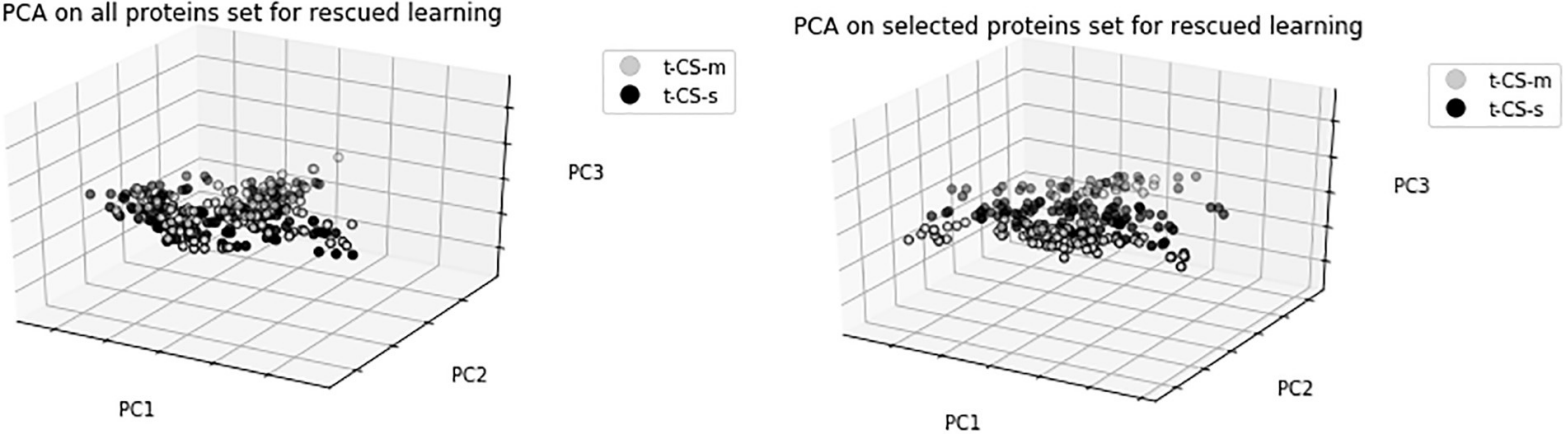
PCA of all proteins set and selected proteins subset for rescued learning.

**Fig 4:**
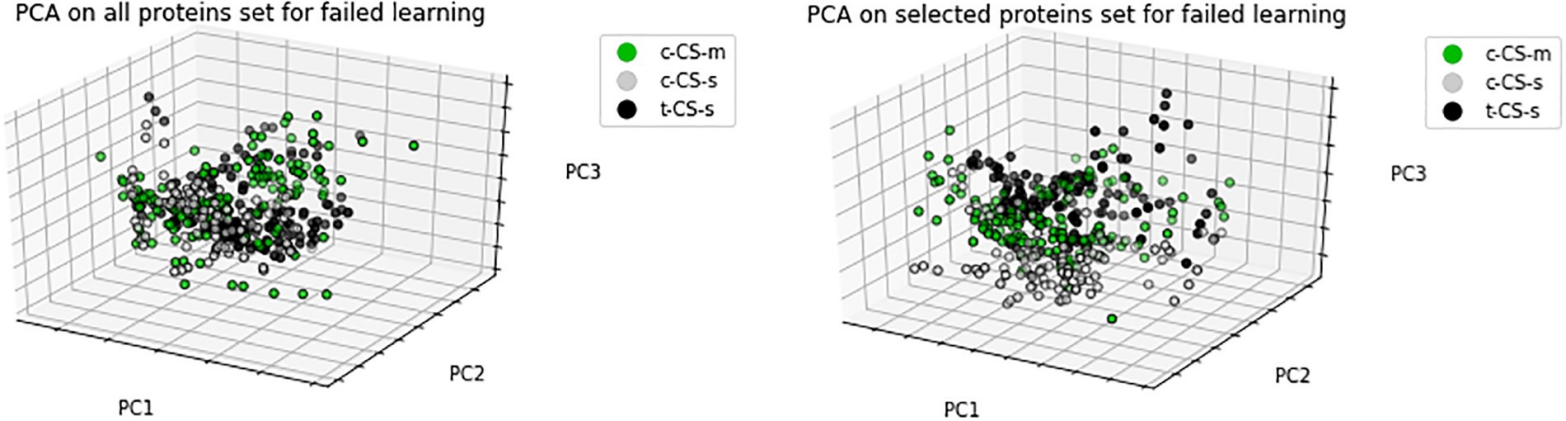
PCA of all proteins set and selected proteins subset for failed learning.

In this section, we conducted PCA for both selected protein subsets and original protein sets for three cases; successful learning, rescued learning and failed learning. We projected these feature sets into 3D spaces in order to validate the selected protein subsets specified in Feature Selection Results. As shown in Figs 2, 3 and 4, the PCA of selected protein subsets can better discriminate the class of mice instances when compared with the PCA of the original protein sets for three cases.

Fig 2 shows the PCA of successful learning. In this case, there are four classes which are normal genotype of control mice with and without memantine and with and without CFC stimulation. As seen in Fig 2, classes are better discriminated with selected proteins.

Fig 3 shows the result of PCA for rescued learning case. In this case, there are two classes which are trisomic mice exposed to CFC with and without memantine. It can be seen that better discrimination of classes can be done with selected proteins.

Fig 4 shows the result of PCA for failed learning. In this case, there are three classes which are trisomic mice exposed to CFC without memantine and normal mice with CFC simulation with and without memantine. Fig 4 also shows better discrimination of classes with selected proteins.

### Classification results

After determination of the different feature subsets for the three cases, classification is performed for differentiating mice in different classes. DNN, gradient boosted tree, random forest and SVM classification methods are executed by using Python and Scikit learn package [41]. Parameters of classifiers are determined based on the grid search hyper-parameter optimization technique which is useful in computational biology problems. With the grid search method, the most suitable parameters for different classification methods are found. In addition, five fold cross validation is applied for preventing overfitting. Together with grid search method, cross validation affects classification accuracy in a positive manner.

The accuracy results of feature subsets shown in the previous Feature Selection Results part are compared with the Higuera’s presented accuracy results.

Table 7 shows the classification accuracies of feature subsets selected in our work and Higuera’s work [25] for successful learning. It can be seen that our feature subset gives higher accuracy results for all classification techniques. For example, while the accuracy of Random Forest was 0.902, it is increased to 0.963. Also, it is observed that the highest accuracy is obtained with SVM with a value of 0.981.

**Table 7:** Accuracy result comparison of normal learning.

	Accuracy Result of Our Work	Previous Work Accuracy Result [25]
Deep Neural Network	0.972	0.967
Gradient Boosted Tree	0.935	0.902
Random Forest	0.963	0.902
SVM	0.981	0.961

Table 8 shows the comparison of rescued learning classification results. The accuracy results of our feature subset are higher than previous work for all classification methods. For example, the accuracy of Random Forest is increased by % 6.3 from 0.883 to 0.946. For rescued learning, the highest accuracy is achieved with DNN and SVM with a value of 0.971.

**Table 8:** Accuracy result comparison of rescued learning.

	Accuracy Result of Our Work	Previous Work Accuracy Result [25]
Deep Neural Network	0.971	0.954
Gradient Boosted Tree	0.933	0.892
Random Forest	0.946	0.883
SVM	0.971	0.921

Table 9 shows the comparison of classifications for failed learning. Similar to previous case, DNN and SVM give highest accuracy results with a value of 0.926. In addition, classification results of our feature subsets are again higher than previous work for all classification methods implemented.

**Table 9:** Accuracy result comparison of failed learning.

	Accuracy Result of Our Work	Previous Work Accuracy Result [25]
Deep Neural Network	0.926	0.921
Gradient Boosted Tree	0.879	0.844
Random Forest	0.892	0.859
SVM	0.926	0.910

## Discussion

Pharmacotherapies of ID are largely unknown as the abnormalities at the complex molecular level which causes ID are difficult to understand. DS which is the prevalent reason of ID and caused by an extra copy of the Hsa21 has been investigated on protein levels. Due to the increase in trisomic genes, protein expression levels of corresponding genes are elevated. Furthermore, in addition to expression of genes on 21 chromosome, protein coding genes on other chromosomes play important roles in DS. Thus, understanding the abnormalities in the protein expressions are very important for developing drugs to rescue learning. For this reason, critical roles of proteins have been analyzed by comparing protein expression levels of normal mice and trisomic mice which are exposed to CFC with or without memantine treatment. In order to find critical proteins in DS, statistical analysis and machine learning methods are used.

In our work, we implemented forward feature selection technique for selecting protein subsets and applied DNN, gradient boosted tree, SVM and random forest classifiers to classify mice more accurately. The classification accuracy results of selected proteins are compared with Higuera et al work [25] in which SOM was applied for clustering of protein based on the similarities in their expression levels and Wilcoxon rank test was done for identifying significantly different protein levels between clusters.

Higuera et al [25] implemented SOM for three cases, successful learning, rescued learning and failed learning, respectively. In the first case, four classes of control mice protein profiles were analyzed for understanding critical proteins in successful learning. In the second case, trisomic mice which are exposed to CFC with or without applying drug memantine were investigated to understand rescue performance of memantine on trisomic mice learning capability. In the last case, using control and trisomic mice, protein profile patterns were analyzed for understanding important factors in learning impairment. They reduced feature subsets from 77 proteins to 11 proteins, 9 proteins and 10 proteins for the three cases, respectively. In this work, we applied naive Bayes classification technique in forward feature selection method rather than SOM which is the clustering technique to group protein levels. We constituted our feature subsets with the same number of proteins selected in Higuera’s work [25] in order to compare the results effectively.

Before selecting feature subsets, the preprocessing step consisted of filling missing part and then normalization is carried out. Missing values are replaced by the average protein expression level of corresponding sample in the same class. This replacement is different from previous works where missing values were handled by the mean value of protein expression levels in same class mice. 15 tissue samples that are three replicates of a five-point dilution series were obtained per mouse. This dilution ratio affects expression level of proteins. Thus, we considered this effect and applied a different technique described in data processing part to handle missing values. For normalization, Z score normalization rather than max-min normalization which is used in Higuera’s work [25] is applied to prevent higher influence of proteins with higher values on the classification outcome.

After preprocessing steps, the forward feature selection algorithm is used to select feature subsets for three cases and these feature subsets are compared with Higuera et al work [25]. For learning process, naive Bayes learner which has been applied to multiclass classification problem is used. In spite of the underlying simplifying assumption of conditional independence, naive Bayes performs well with more than two classes problem. These distinct preprocessing and feature selection methods affected results in a good way and important proteins that were not highlighted in the previous works are found.

Critical proteins in DS have been found to be related with different pathways and processes, such as MAPK and MTOR pathways, immediate early genes (IEGs), AD, neurotrophin signaling pathway and apoptosis. In our work, in addition to these pathways and processes, we evaluated proteins according to their relations with immune system. It was hypothesized that DS causes an increase in interferon signaling which triggers the protective defenses of the immune system [26]. Thus, we searched proteins for finding a connection between L/M and immune response.

Eleven proteins were found to be significantly different in successful learning case: SOD1, ubiquitin, pGSK3B, S6, CaNA, IL1B, BAX, pNR2A, BDNF, pJNK and pCFOS. These proteins play important roles in L/M, immune response, MAPK pathyway, mTOR pathway and AD. When compared with Higuera’s work [25], 4 proteins (SOD1, pGSK3B, S6 and CaNA) are found to be common. Three of the proteins (SOD1, pGSK3B and CaNA) are related to immune system.

SOD1 found on chromosome 21 causes immune abnormalities in Amyotrophic lateral sclerosis (ALS) disease [48] and increases reactive oxygen in DS [49]. Ribosomal Protein S6 and pGSK3B are components of mTOR pathway which takes action in learning [50]. Also, in the literature it is noted that GSK3 inhibitors provide to prevent excessive inflammation and ameliorate the autoimmune disease [51]. CaNA and IL1B are known to be pathogenesis of AD [52, 53]. In addition, it is known that IL1B is natural suppressor of innate inflammatory [54]. BAX and ubiquitin play critical roles in apoptosis and immune response [55, 56]. BDNF takes action in L/M [57] and bridges inflammation and neuroplasticity [58, 59]. pNR2A has well established roles in learning [60]. pJNK is component of MAPK pathway which is associated with L/M [61]. pCFOS is an IEG and is important in long term memory and neurological function seen in AD [62]. In the first case, by analyzing protein expression levels of control group mice, it can be deduced that proteins which are related to the L/M pathway and the immune responses are critical in successful learning.

Nine proteins are highlighted in the rescued learning case: BRAF, S6, CDK5, BDNF, pCREB, PKCA, SOD1, PSD95 and pNR2A. Two out of nine proteins are common with Higuera’s work [25]. Four of these proteins (S6, BDNF, SOD1 and pNR2A) are also found in successful learning case and their importance is explained above. BRAF and PKCA are associated with MAPK pathway and important in learning [63, 64]. CDK5 is synaptic protein and plays a critical role in long-term memory [65]. Also, it regulates the evasion of tumors from the immune system [66]. PSD95 is a neuropathological marker of AD observed in later stage of DS [67]. In addition, PSD95 colocalizes with major histocompatibility complex class I (MHCI) which is the signature of its expressed proteins and is important for the immune system to differentiate self from nonself [68]. CREB regulates crucial cell stages and participates in neuronal plasticity [69]. Thus, it can be concluded from the second case that proteins which are important in rescued learning are relevant to the L/M and the immune response.

In the case of failed learning, ten proteins are found to be significant: p38, pPKCAB, CAMKII, pCAMKII, GluR3, DSCR1, nNOS, BAX, pCFOS and ERK. Two of these proteins (BAX and pCFOS) were also highlighted in successful learning and described above. The remaining selected proteins are largely connected to MAPK signaling pathway, such as P38, pPKCAB, CAMKII, pCAMKII and ERK. GluR3 is related to glutamate receptors which cause memory deficit if excess amount of glutamate binds to receptor [70]. DSCR1 is known to be over expressed in DS and affects signaling pathway [71]. Failed learning case also shows us the importance of signaling pathway in the learning process.

PCA is also done for both selected protein subsets and original protein sets for the three cases; successful learning, rescued learning and failed learning. It is shown that selected protein subsets can better discriminate the class of mice instances when compared with the PCA of the original protein sets for all the indicated cases.

After finding critical proteins for three cases, DNN, gradient boosted tree, random forest and SVM classification methods are applied. The parameters of classifiers are optimized with grid search. Also, 5 fold cross validation is done to prevent overfitting. The accuracy results of our feature subsets are found to be higher than previous work for all classifiers. DNN and SVM achieved the highest overall classification accuracy followed by random forest and then gradient boosted tree.

Multiple layers in a deep learning model can learn features from a wide perspective with higher flexibility. Thus, it is logical to obtain good results with DNN. SVM maps data to a feature space and then classify the data. It explicitly determines the decision boundary directly from the training data. Parameter optimization step is required to build an efficient SVM model. Using grid search method, parameters are selected and SVM with the selected parameters gives higher accuracy results. Accuracy result of random forest is lower than SVM and Deep Neural Network. The reason of lower accuracy can be the size of data as random forest generally needs larger number of instances for performing its randomization concept in a good way. Also, decision trees used as base learners in the random forest cannot exactly learn many of soft linear boundaries at the decision surface which can cause lower success than the SVM non linear boundaries. Gradient boosted tree is prone to overfitting as it tries to find optimal linear combination of trees in relation to given train data. This tuning stage may be the reason of the lowest accuracy obtained by gradient boosted tree.

In conclusion, our work described in this paper provides better learning model and shows that proteins which are found to be related to the L/M and the immune system are critical in successful learning. Therefore, by extracting information from these protein subsets, the effective drugs can be developed for the treatment of DS.

## Supporting information

S1 Dataset Protein expression profiles of 77 proteins obtained from control and trisomic Ts65Dn mice. These data are a subset of those used in Ahmed’s work [27](ZIP).
(ZIP)
